# A Long-Term Study of Young Children's Rapport, Social Emulation, and Language Learning With a Peer-Like Robot Playmate in Preschool

**DOI:** 10.3389/frobt.2019.00081

**Published:** 2019-09-03

**Authors:** Jacqueline M. Kory-Westlund, Cynthia Breazeal

**Affiliations:** MIT Media Lab, Massachusetts Institute of Technology, Cambridge, MA, United States

**Keywords:** children, language development, mimicry, peer modeling, rapport, relationship, social robotics, storytelling

## Abstract

Prior research has demonstrated the importance of children's peers for their learning and development. In particular, peer interaction, especially with more advanced peers, can enhance preschool children's language growth. In this paper, we explore one factor that may modulate children's language learning with a peer-like social robot: rapport. We explore connections between preschool children's learning, rapport, and emulation of the robot's language during a storytelling intervention. We performed a long-term field study in a preschool with 17 children aged 4–6 years. Children played a storytelling game with a social robot for 8 sessions over two months. For some children, the robot matched the level of its stories to the children's language ability, acting as a slightly more advanced peer (*Matched* condition); for the others, the robot did not match the story level (*Unmatched* condition). We examined children's use of target vocabulary words and key phrases used by the robot, children's emulation of the robot's stories during their own storytelling, and children's language style matching (LSM—a measure of overlap in function word use and speaking style associated with rapport and relationship) to see whether they mirrored the robot more over time. We found that not only did children emulate the robot more over time, but also, children who emulated more of the robot's phrases during storytelling scored higher on the vocabulary posttest. Children with higher LSM scores were more likely to emulate the robot's content words in their stories. Furthermore, the robot's personalization in the *Matched* condition led to increases in both children's emulation and their LSM scores. Together, these results suggest first, that interacting with a more advanced peer is beneficial for children, and second, that children's emulation of the robot's language may be related to their rapport and their learning. This is the first study to empirically support that rapport may be a modulating factor in children's peer learning, and furthermore, that a social robot can serve as an effective intervention for language development by leveraging this insight.

## 1. Introduction

Children's early language development is linked to their academic and overall life success. Numerous studies in the United States, for example, have found that children who are not exposed to rich language learning opportunities as they grow up—such as vocabulary-building curricula, cognitively challenging preschool activities, greater numbers of novel words and total words heard—may be significantly impacted, showing language deficits, lower reading comprehension, and lower vocabulary ability (Huttenlocher et al., 1991, 2002, 2010; Hart and Risley, 1995; Fish and Pinkerman, 2003; Griffin et al., 2004; Paez et al., 2007; Snow et al., 2007; Perkins et al., 2013; Schwab and Lew-Williams, 2016). Numerous interventions have been developed to support children's early language development, such as preschool readiness programs, teacher, and parent resources, and a wide range of language-focused educational apps, games, and computer programs.

One way children's language learning can be supported is through peer interaction. Children's peer relationships provide opportunities for openness, exploration, and discovery. Research from the past several decades shows that children's peers, particularly more advanced peers, can enhance their overall preschool competency and language growth (Fuchs et al., 1997; Mathes et al., 1998; Topping, 2005; Schechter and Bye, 2007; Whitebread et al., 2007; Mashburn et al., 2009; Justice et al., 2011; DeLay et al., 2016; Lin et al., 2016). Mashburn et al. (2009), for example, measured preschool children's receptive and expressive language skills at the start and end of a school year. Children's language growth during the year was positively related to their peers' expressive language abilities, a result later replicated by Justice et al. (2011). Notably, children, particularly children with lower skills, appeared to benefit most from having higher ability peers around them.

This research is in line with various theories about how peer learning occurs, including Vygotsky's theory that a child's more advanced peers can help support or scaffold the child in acquiring and practicing skills that are otherwise beyond their skill level (Vygotsky, 1978; Tudge and Rogoff, 1989; Rubin et al., 1998); Bandura and Walters' social learning theory which argues that children frequently learn through observing and imitating others (e.g., observing and imitating their speech; Bandura and Walters, 1963; Bandura, 1971; Rubin et al., 1998); and Piaget's theories regarding the importance of dialogue and discussion among peers in promoting cognitive development (Piaget, 1932; Tudge and Rogoff, 1989; Rubin et al., 1998; De Lisi and Golbeck, 1999).

Because children's peers can significantly and positively affect their language learning, numerous researchers in human-robot interaction have hypothesized that playing with a peer-like robot companion may lead to similar benefits. For example, some robots have been positioned as slightly advanced peers (e.g., Kanda et al., 2004; Kory and Breazeal, 2014; Gordon et al., 2016; Kory Westlund et al., 2017b); while others have been positioned as younger peers or novices (e.g., Movellan et al., 2009; Tanaka and Kimura, 2009; Tanaka and Matsuzoe, 2012; Gordon and Breazeal, 2015; Hood et al., 2015; Tanaka et al., 2015). Some virtual agents have also been created as peer-like learning companions (Bers et al., 1998; Cassell and Ryokai, 2001; Ryokai et al., 2003; Cassell, 2004; Cassell et al., 2007). In language learning applications, research has focused primarily on children's vocabulary learning, often in English and often with English as a second language, though language production is also a growing area of study (Kanero et al., 2018).

It is also very common for robots to be situated as teachers or tutors (e.g., Robins et al., 2005; You et al., 2006; Chang et al., 2010; Lee et al., 2011; Alemi et al., 2014; Serholt et al., 2014; Deshmukh et al., 2015; Kennedy et al., 2016; Park et al., 2017b; Vogt et al., 2017, 2019; Rintjema et al., 2018). A recent survey of 101 studies of social robots in education revealed that 86% of studies set up robots as teachers or tutors, 4% positioned the robot in a mixed tutor/teacher role, only 9% set up the robot as a peer or novice, and 1% gave the robot another role (Belpaeme et al., 2018). In this survey, nearly 60% of the studies surveyed involved children, and it included studies of many different educational activities, including language, math, and reading.

Given this interest in using social robots to support children's language learning, we should examine more closely what modulates children's learning with peers, and by extension, mechanisms that robots can use to be more effective learning companions. That is: are children's peers approximately equal as sources for promoting language learning, or will children learn more effectively from some peers than from others? What features or behavior might help a social robot better enable children's language learning?

Some work has begun exploring these questions. For example, robots that use nonverbal social cues and nonverbal immediacy behaviors have led to increases in children's engagement, learning, and relationships during educational activities (e.g., Kanda et al., 2004, 2007, 2012; Breazeal et al., 2016; Kennedy et al., 2017; Kory Westlund et al., 2017a,b). These results jibe with literature in psychology and education, where research has linked improved learning outcomes to use of appropriate social cues (e.g., Bloom, 2000; Meltzoff et al., 2009; Sage and Baldwin, 2010; Kuhl, 2011), social interaction and greater numbers of conversational turns (e.g., Hoff, 2006; Romeo et al., 2018a,b), and nonverbal immediacy (Mehrabian, 1968; Christophel, 1990; Witt et al., 2004). Robots that personalize content or behavior to children have also led to increased learning and engagement (e.g., Leite et al., 2012; Kory and Breazeal, 2014; Gordon et al., 2016; Palestra et al., 2016; Scassellati et al., 2018; Park et al., 2019).

Another mechanism that may improve children's learning is rapport, as suggested by two recent studies of children's language learning during storytelling with social peer-like robots (Kory Westlund et al., 2017b; Kory-Westlund, 2019; Kory-Westlund and Breazeal, 2019b). Kory Westlund et al. (2017b) found that playing with a robot with a more expressive voice led to increases in children's engagement and vocabulary learning as well as increased emulation of the robot's language. Kory-Westlund (2019) found that children's language emulation, positive emotion, and acceptance of the robot were positively affected by the robot's use of speech entrainment and an appropriate backstory about its abilities. These studies suggest that children's rapport may be reflected in their language emulation, a result that jibes with related work showing that humans who have greater rapport with each other will mimic each other's language (e.g., Niederhoffer and Pennebaker, 2002; Pennebaker et al., 2003; Huttenlocher et al., 2004; Tausczik and Pennebaker, 2010; Ireland et al., 2011; Babcock et al., 2014) and vocal prosody (e.g., Porzel et al., 2006; Reitter et al., 2011; Borrie and Liss, 2014) more.

Earlier work with adults and robots (Kidd and Breazeal, 2008; Lubold et al., 2016, 2018; Lubold, 2017), as well work in human-human tutoring (Sinha and Cassell, 2015a,b), have also suggested links between learning and rapport. Children's social bonds with their teachers can predict their performance (Wentzel, 1997). Children who have stronger parasocial relationships with media characters may learn more effectively from those characters (Gola et al., 2013; Richards and Calvert, 2017).

Taken together, the research so far suggests that children's rapport with an interlocutor may affect their learning and language behavior. However, these studies were primarily one session; they did not examine children's learning or language behavior over time. As such, one open and important question was whether children would emulate the robot's language long-term, and if they did, whether this would be related to their vocabulary learning or their rapport with the robot. To explore this question, we performed new analyses on an existing dataset from an 8-session study in which children played a storytelling game with a peer-like social robot. The design and early results from this study were presented in (Kory, 2014; Kory and Breazeal, 2014; Kory Westlund and Breazeal, 2015); here we present the full methodology, as well as results and discussion.

## 2. Methodology

### 2.1. Research Questions

We wanted to explore connections between children's learning, their rapport, and their emulation of a peer-like robot's language behavior. We asked whether children would be more likely to emulate language of a robot with whom they had more positive rapport, whether this was correlated with their learning, and furthermore, whether children's emulation or rapport were consistent over time.

### 2.2. Design

**Figure 1:**
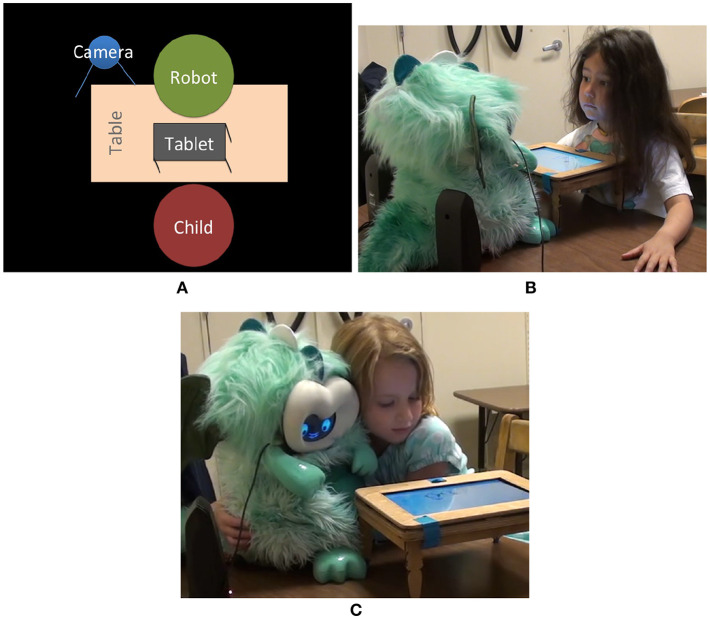
**(A)** The robot was placed on a table across from the child. The tablet was set in a small table between them. The camera was set up behind the robot to the left. **(B)** A girl listens while the robot tells a story. **(C)** This girl turned the robot and tablet so she could sit beside the robot. Written informed consent was obtained to use these images.

We performed new analyses on an existing dataset that included stories from 14 children, who had played a storytelling game with a robot 1–2 times per week for 8 sessions (Figure 1) (Kory, 2014; Kory and Breazeal, 2014; Kory Westlund and Breazeal, 2015).

The original study explored whether a peer-like social robot could facilitate preschool children's oral language development. In addition to being one of the first studies exploring the effectiveness of a long-term, storytelling intervention, this study examined whether personalizing the general language complexity of the robot's stories might increase children's learning of new words and use of more complex language in their own stories. The hypothesis was that presenting stories of an appropriate challenge for the child, slightly ahead of the child's general ability in the zone of proximal development, might promote learning (Vygotsky, 1978; Csikszentmihalyi, 1990). Thus, the study followed a two-condition design.

Two versions of each story told by the robot were created, a harder version and an easier version (for more detail regarding story creation, see Kory, 2014; Kory and Breazeal, 2014). In the first half of the study (sessions 1–4), all children heard the same versions of the stories. In the second half of the study (sessions 5–8), children in the *Matched* condition (12 children—6 female, 6 male) heard stories matched to their language ability (i.e., harder stories for children with higher ability; easier stories for children with lower ability. Children in the *Unmatched* condition (5 children—4 female, 1 male) heard stories that were not matched (e.g., easy stories for children with higher ability).

### 2.3. Participants

Seventeen children aged 4–6 years (10 female, 7 male) from two Boston-area preschools (9 from the first and 8 from the second) participated in the original study. Children were recruited from two schools in order to recruit sufficient children for the study. There were three 4-year-olds, thirteen 5-year-olds, and one 6-year-old (*M* = 4.88, *SD* = 0.49). The 6-year-old girl did not complete the final session, and one 4-year-old girl completed only the first 4 sessions. Children in this age range were targeted because their expressive language abilities are developed enough to be able to tell stories. They are still in the process of developing their narrative abilities. Younger children, as was discovered during pilot testing, may not tell stories at all and are less likely to understand and follow the rules of the game.

For the purposes of our analyses here, our data included 206 stories from 14 children (8 female, 6 male, two 4-year-olds, twelve 5-year-olds, age *M* = 4.86, *SD* = 0.36) and full transcripts from all 17 children (3 children did not tell stories).

Children's parents gave written informed consent prior to the start of the study, and all children assented to participate. The protocol was approved by the MIT Committee on the Use of Humans as Experimental Subjects.

### 2.4. Hypotheses

We expected the following:

- **H1:** Children who showed greater rapport with the robot would be more likely to learn the target vocabulary words, with receptive knowledge indexed by vocabulary assessment scores and productive knowledge by use of the words in their stories. We expected this because prior work has shown that rapport can facilitate learning (Sinha and Cassell, 2015a,b), and children have previously mirrored a robot's vocabulary in their stories (Kory Westlund et al., 2017b)
- **H2:** Children who showed greater rapport would be more likely to emulate the robot's language in their stories and throughout the full interaction session. We expected this because people frequently mirror the language and behavior of those with whom they have rapport (e.g., Dijksterhuis and Bargh, 2001; Niederhoffer and Pennebaker, 2002; Huttenlocher et al., 2004; Chartrand and van Baaren, 2009; Tausczik and Pennebaker, 2010; Ireland et al., 2011; Babcock et al., 2014).
- **H3:** Because of the expected connections between children's rapport and their learning, we also expected that children who emulated the robot's language more would also show more vocabulary learning.
- **H4:** We expected children's rapport and their emulation of the robot's language to increase over time as they became more familiar and comfortable with the robot.
- **H5:** Children who heard personalized stories from the robot would emulate more, learn more words, and have greater rapport. We expected this because of suggested links between a robot's personalization and children's engagement and learning (e.g., Leite et al., 2012; Gordon et al., 2016; Palestra et al., 2016; Scassellati et al., 2018; Park et al., 2019).

### 2.5. Procedure

Each child participated in a pretest session and 8 sessions with a teleoperated robot, over 10 weeks (Kory, 2014; Kory and Breazeal, 2014; Kory Westlund and Breazeal, 2015). During the pretest, children were given a language assessment, a subset of the Preschool Language Scale, 5th Edition (Zimmerman et al., 2011), to assess aspects of their expressive and receptive language ability. This assessment did not use any of the robot's target words. Children were also given a separate receptive vocabulary pretest for the target words the robot used in its stories. In this test, for each word, children were shown a set of four pictures and were asked to point to the picture showing the target word.

These initial assessments was used to split children into two groups: higher language ability (above the mean), and lower language ability (below the mean). These categorizations were for this study only; “higher/lower language ability” did not mean children were necessarily above or below what might be expected for their age, just that they were divided into two groups for the purposes of the robot's language level personalization. Children were randomly assigned to the *Matched* or *Unmatched* conditions after these assessments; their initial language assessment scores were taken into account in an attempt to balance language ability across conditions.

Each of the 8 sessions with the robot was 10–15 min long (Figure 1). The robot briefly engaged the child in conversation (e.g., asking if the child had done anything fun that morning or sharing a fact about itself), then showed a story scene on a tablet and told a short story. Next, the child was invited to tell their own story about the scene. The robot then showed a second story scene and told a second story, and the child was invited to tell a second story. After a brief closing conversation, the interaction ended. In some sessions, the robot showed a story scene but asked the child to tell a story first. If children declined to tell their own story, the robot briefly encouraged them to do so, but if they refused again, the robot moved on.

As mentioned above, in the first half of the study (sessions 1–4), all children heard the same stories. In the second half of the study (sessions 5–8), children in the *Matched* condition heard stories matched to their language ability, while children in the *Unmatched* condition heard stories that were not matched.

A storytelling activity was used to promote language development because storytelling is a socially situated activity that combines play and narrative, which are two important aspects of children's learning and development (Nicolopoulou, 1993; Engel, 1995). Storytelling can allow collaborative, creative conversation and language practice, and can support emergent literacy skills, including metalinguistic knowledge about language patterns, structure, and function; vocabulary; “decontextualized” language that can be understood outside its original context; as well as supporting cognitive, communicative, and linguistic development more broadly (Engel, 1995; Cassell, 2004; Curenton et al., 2008).

Children were interviewed about their perception of the robot and interaction after sessions 4 and 8. The questions were adapted in part from (Jipson and Gelman, 2007; Kahn et al., 2012). Children were invited to answer numerous questions using a verbal 3-point scale (“a lot,” “a little bit,” or “not very much”). While this methodology presents some challenges due to children's tendency to answer in socially acceptable ways, anecdotally, children's engagement and interest observed during the activities was reflected in their interview responses. Furthermore, many of the interview questions were followed up by asking children to explain their response or to say more, which helped give context to children's ratings. All interview questions and language assessments are available on figshare at https://doi.org/10.6084/m9.figshare.8144456.

### 2.6. Materials

#### 2.6.1. Robot

This study used the Dragonbot (Setapen, 2012; Kory et al., 2013) as the learning companion. This robot is capable of expressive movement based on “squash and stretch” principles of animation. It can display a variety of facial expressions on the smart phone that also runs its software, as well as play sounds or speech. The robot wore green fur, was named “Green,” and was referred to in a distinctly non-gendered way by the experimenter throughout the study.

The robot followed a script of speech, expressions, and movement. Speech was recorded by a human adult female. The pitch of the speech was shifted higher to sound more like a child.

#### 2.6.2. Teleoperation

A human operator used a custom control interface to send action and speech commands to the robot. The teleoperator attended to the child's speech and actions in order to trigger the robot's actions (e.g., playing back speech or showing a facial expression) at appropriate times. Including a human in the loop allowed the robot to appear autonomous while sidestepping technical barriers such as autonomatic speech recognition and natural language understanding. When the robot's actions depended on what the child said or did, such as during the introductory conversation or when asking the child if they wanted to tell a story, the teleoperator selected among a limited set of dialogue options. The robot's gaze was automatically directed to either look up at the child or down at the game, based on data collected during the pilot study regarding where children look during play.

The teleoperator followed several general rules. First, the teleoperator made the robot's behavior as socially contingent as possible—reacting to the child as closely to as a human would in the same circumstance. When the child spoke, the robot would acknowledge through speech, verbal exclamations such as “Ooh!” and “Oh no!,” smiles, and short affirmative non-linguistic noises. These acknowledgments were primarily triggered during pauses in the child's speech. The same sounds or animations were not triggered twice in close succession, though the same sounds and animations were often used multiple times per session. Finally, the teleoperator made the robot's behavior as consistent as possible across participants, using the same set of sounds and animations with approximately the same frequency for all children. The same person operated the robot for all participants and had been previously operated this robot in numerous earlier studies.

#### 2.6.3. Storytelling Game

The storytelling game was inspired by the game developed by Ryokai et al. (2003) for their virtual peer, in which the virtual agent which took turns with children telling stories about characters in a toy castle. In this study, the shared game surface was a tablet screen set into a small wooden table. Story scenes showed a background image with several characters and objects that could be dragged around on the screen, much like virtual stick puppets. When the robot told stories, the characters were moved automatically in concert with the robot's speech. These movements were recorded and played back so that they would be consistent for all children. There were no additional animations or sound effects.

**Figure 2:**
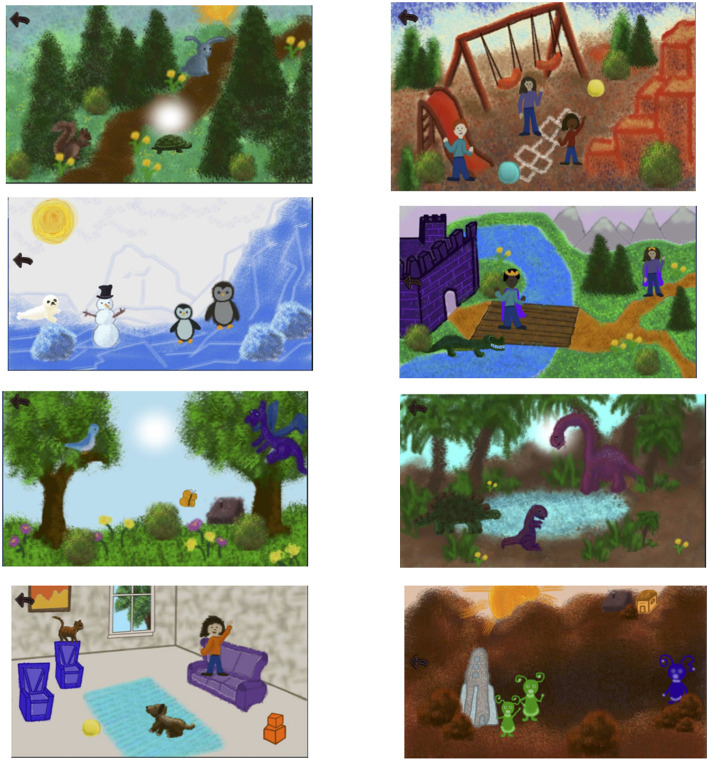
The eight story scenes used for the storytelling game. Two stories were written for each scene, for a total of 16 stories.

The game included eight story scenes (Figure 2). Over the course of the study, the robot told two stories using each scene.

The robot's stories were based on stories told by children during pilot testing of the game at the Boston Museum of Science (Kory, 2014). Two versions of each story were crafted for the personalization with the same general content, but with one having greater with greater language complexity (“hard” stories) and one with less (“easy” stories Kory and Breazeal, 2014). For example, part of one easier story included, “George liked to climb up massive icebergs. He liked to slide back down in the snow,” while the more complex version was, “George enjoyed climbing up to the very top of massive icebergs, then sliding all the way back down on his belly, beak first”.

#### 2.6.4. Vocabulary

Twenty-four target vocabulary words were selected from Andrew Biemiller's “Words Worth Teaching” lists (Biemiller, 2010), including nouns (e.g., structure, clump), verbs (e.g., expect, plunge), and adjectives (e.g., ancient, massive). Three words were used in each of the robot's stories. Because the robot told two stories each session, six words were used each session. After sessions 1–4, all the words had been introduced. During the sessions 5–8, the words were used again in new stories to provide additional opportunities for learning. Children were tested on the vocabulary words using a picture-based assessment before and after the study. In each item on the assessment, children were shown four pictures. They were asked to point to the picture corresponding to the target word.

### 2.7. Data

Audio and video of the study sessions were recorded with a camera beside the robot (Figure 1). Children's responses to the vocabulary assessments and interview questions were recorded on paper and later transferred to a spreadsheet.

### 2.8. Data Analysis

The recorded audio was used to transcribe children's speech. Children's stories were extracted from the full transcripts. All children spoke during the conversations with the robot, and most told stories as well.

The data we analyzed in this paper included 206 stories from 14 children and full transcripts from 17 children (3 children did not tell stories). In these data, we examined children's use of key vocabulary words and key phrases used by the robot, children's emulation of the robot's stories during their own storytelling, and children's language style matching (LSM). LSM is a measure of overlap in function words and speaking style as opposed to content words. Our phrase matching metrics looked primarily at content words. Research has shown that the more “in sync” two people are, the more they will match function words in their speech; it may reflect rapport and relationship (Niederhoffer and Pennebaker, 2002; Pennebaker et al., 2003; Tausczik and Pennebaker, 2010; Ireland et al., 2011; Babcock et al., 2014). We use LSM here as a measure of rapport.

One limitation of this methodology is that LSM is a linguistic measure of rapport. It would be useful in future work to examine additional ways of measuring children's rapport with the robot, to see whether children's word and phrase use was related to any non-linguistic signs of rapport or relationship as well.

#### 2.8.1. Target Words and Key Phrases

Using automated software tools, we counted the number of times children used each of the target vocabulary words in each session and in their stories. This analysis was performed on the full transcripts of each session. Usage of the words may reflect expressive vocabulary ability, which is often a stronger indicator of knowledge of a word than the receptive knowledge tested with the vocabulary assessment (Bloom, 1974; Ingram, 1974; Sénéchal, 1997), as well as mimicry of the robot. We also counted the number of times children used key phrases that the robot had used (e.g., “Once upon a time,” “I'll tell a story about…,” “See you later, alligator!”). For these, our goal was to see whether children adopted any of the robot's frequently used phrases, as this mimicry may reflect greater rapport.

#### 2.8.2. Language Style Matching (LSM)

LSM analysis requires a minimum of 50 words per participant in the conversation, but works better with a greater number of words (Pennebaker et al., 2003; Tausczik and Pennebaker, 2010). Thus, to get sufficient data for an LSM analysis, we aggregated all of each child's stories for sessions 1–4 (the first half of the study) and then for sessions 5–8 (the second half). We obtained an LSM score for each set using software tools to access the Receptivity API (Tausczik and Pennebaker, 2010). LSM scores range from 0 to 1.00, but more often range from 0.5 to 1.00. The closer the score is to 1.00, the more matching is present.

#### 2.8.3. Stories and Phrase Matching

We analyzed children's transcribed stories in five ways: length (in seconds), word count, vocabulary word use, and emulation of the robot's phrases. We created an automatic tool to obtain phrase matching scores comparing each child story to each robot story that the child had heard prior to telling the story. For example, a story told by a child in session 2 was compared to the stories the robot told in session 1 as well as any stories the robot told before the child in session 2. The analysis was then threefold: (1) compare each child story to the robot story just prior to it; (2) compare each child story to other stories in the same scene; (3) compare each child story to all stories prior to it. The matching algorithm was as follows:

1. Remove stopwords (i.e., words with no significant information such as “the,” “uh,” and “an”).
2. Stem words, i.e., convert words to their original form (e.g., “running” becomes “run”).
3. Find all N-grams in each text, where an N-gram is a continuous sequence of N words from the text.
4. Remove duplicate N-grams from one text.
5. Count how many N-grams are the same in both texts.
6. Return that number as the match score.

This produced a score reflecting the number of exact matches—i.e., words used in the same order by both the child and robot. It also produced a higher match score for texts that have both more matching phrases and longer matching phrases. We also implemented an algorithm for counting similar matches that were close to each other, but not exactly the same. This algorithm followed the same steps listed above, where step 5 (counting matching N-grams) used a fuzzy string matching algorithm to determine if N-grams matched.

For exact matches, we used *N* = 3 because a smaller *N* may not retain enough information to be considered actual phrase matching, while a larger *N* may contain more information than would comprise a single phrase. For similar matches, we used *N* = 4, so that when phrases differed by one or two words, they might still match.

For example, one of the robot's stories included the sentences, “But Turtle still couldn't find Squirrel. Eventually, it got dark out and they all got sleepy. So Squirrel had to show his hiding place.” After stopword removal and stemming, this was converted to: “turtle still couldn't find squirrel eventually get dark out they all get sleepy squirrel show hiding place.” One child's story included the similar section, “But he still couldn't find Squirrel. Then he bumped into him and started playing. And it's getting late out. So Squirrel had not showed his hiding place,” which was converted to “he still couldn't find squirrel then he bump into him start play get late squirrel show hiding place.” This segment included several exactly matching phrases, e.g., “couldn't find squirrel,” as well as several similar matching phrases, e.g., (robot) “squirrel show hiding place” \ (child) “late squirrel show hiding.”

## 3. Results

First, we discuss children's vocabulary learning and information about the kinds of stories children told. Some of these results were previously reported in Kory (2014); Kory Westlund and Breazeal (2015); Kory-Westlund (2019). We also briefly discuss children's responses to the interview questions about their perception of the robot. These interviews are relevant because they showed that nearly all children reported liking the robot, and that children's liking was not identical with our measures of emulation and rapport.

Next, we present our new analyses regarding children's use of the target words and key phrases, emulation of the robot, LSM scores, and correlations among these measures. Because the new analyses were *post-hoc*, we corrected for multiple comparisons using the Benjamini Hochberg method (to control the false discovery rate), which indicated that the results with *p* < 0.011 could be considered significant.

### 3.1. Interviews

**Figure 3:**
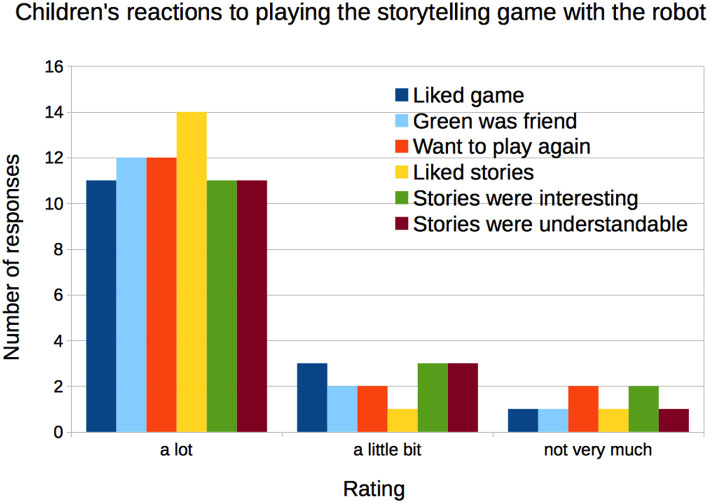
The majority of children reported liking the robot and the storytelling game.

As reported in Kory (2014), most children reported that they liked the game a lot (76.5%), that the robot was their friend (87.5%), that they wanted to play again (87.5%), that they liked the stories (93.6%), and that they thought the stories were interesting (93.6%), and understandable (93.6%) (Figure 3). There were no differences by condition.

### 3.2. Target Vocabulary

**Figure 4:**
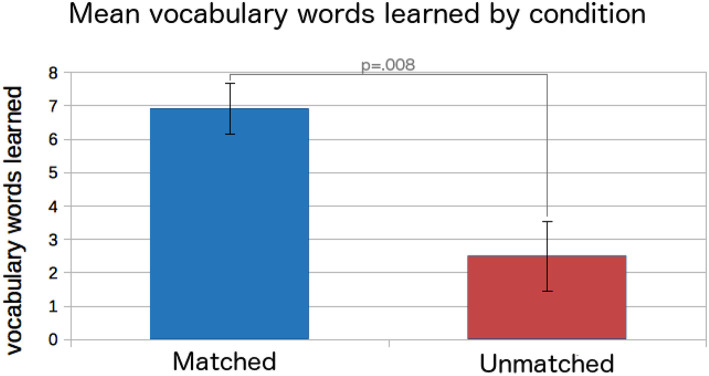
Children's vocabulary scores increased over the study, but more so in the *Matched* condition.

Across all the children, children's scores on the vocabulary assessment increased from the pretest (mean words correct = 13.4 of 24, *SD* = 3.62) to the posttest (*M* = 18.9, *SD* = 2.84), *t*_(14)_ = 7.21, *p* < 0.001, *d* = 1.7. Children's scores increased by a mean of 5.7 words (*SD* = 3.08). Children's scores increased more in the *Matched* condition (*M* = 6.91 more words correct at the posttest, *SD* = 2.51) than in the *Unmatched* condition (*M* = 2.50, *SD* = 2.08), *t*_(13)_ = 3.13, *p* = 0.008, *d* = 1.9 (Figure 4).

### 3.3. Stories

Nine children told stories aloud every session. Five children told primarily silent stories, in which they spent time dragging characters on the tablet and sometimes murmuring to themselves, but not speaking aloud very often. Their stories often appeared short because only spoken words were counted. Several of these “silent tellers” began vocalizing their stories more by the final session, telling stories closer in length to the other children. Three children told no stories, though they did talk at other times.

The children who spoke aloud told 206 stories with a mean word count of 81.7 words (*SD* = 77.8). Of these, 141 stories were 20 words or longer; the shorter stories were primarily from the children who only occasionally spoke while playing the storytelling game.

Qualitatively, children covered a range of themes in their stories. We observed that children often borrowed elements from the robot's stories—such as character names and activities characters performed. For example, one of the robot's stories was about a boy named Micah, who played ball with his friends. One child continued using this name and theme (XX's indicate inaudible words in the transcript):

“One time there were three friends, XX, Micah and Isabella. Micah liked going on the swings. Isabella liked going on the slide. One time they made a new friend, Daisy. She liked ball. One time she hid behind a bush until nobody saw her. Then both of the kids that were playing, approached and hid. Then, Micah slid down the slide and saw her. She stepped out but landed on the top of the brick tower. So then, they both came down together. The end.”

Several children also retold versions of the robot's stories, without prompting (they were merely asked to tell a story and were not prompted with regards to content). For example, after the robot told a story about three animals that played hide-and-seek together, one child told the following story:

“Once upon a time there was a squirrel named, Squirrel, a turtle named Turtle and a rabbit named Rabbit. That particular day they played hide and seek. Squirrel hid in the mud. Turtle hid in the trees while Bunny counted. One, two, three, four. Found you! Found you, Turtle. My turn. XX behind a tree. Squirrel found Turtle. And then they played again and again. The end.”

Our observations of these emulations suggested that children were, in fact, emulating the robot's stories, which was revealed quantitatively in our language eumulation results below.

### 3.4. Keywords and Key Phrases

**Figure 5:**
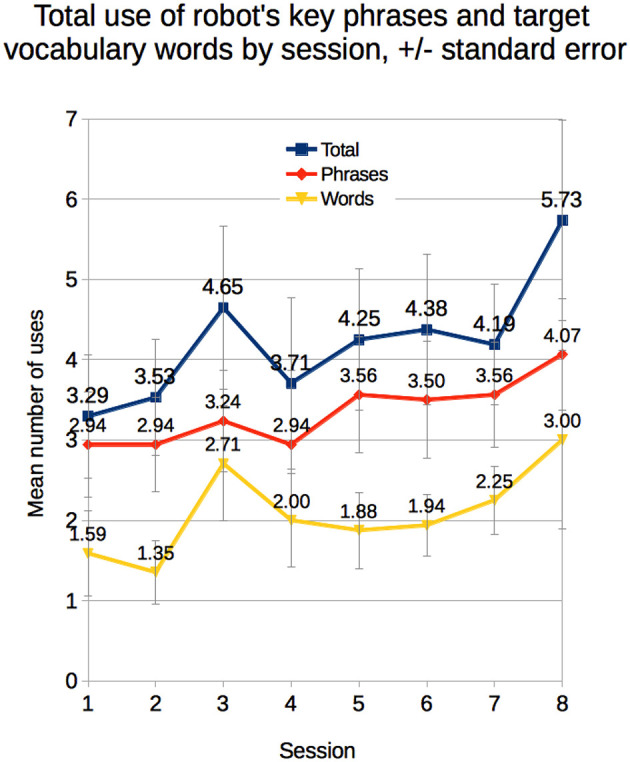
Children's mean use of the robot's key phrases and target vocabulary words by session.

We performed mixed analysis of variance with condition (between: *Matched* vs. *Unmatched*) and mean of sessions (within: sessions 1–4 vs. sessions 5–8) on children's use of the robot's target vocabulary words and key phrases. We observed a trend toward a main effect of session on the total number of key phrases and target vocabulary words children used from the first half to the second half of the study, *F*_(1, 13)_ = 2.95, *p* = 0.11, *d* = 0.22 (Figure 5). Children used somewhat more of the key phrases and target words in the second half of the study than in the first half. In particular, children tended to use the phrases “once upon a time” and “See you later, alligator” more in later sessions.

### 3.5. LSM

**Figure 6:**
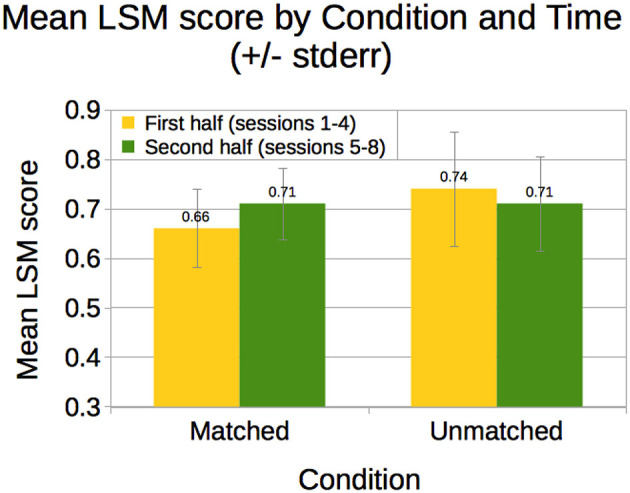
Children's mean LSM scores by condition for the first half vs. second half of the study.

We observed LSM scores ranging from 0.063 to 0.892, with a mean of 0.696 (*SD* = 0.212). Only two children had scores below 0.500; in both cases, their scores increased from the first half to second half of the study. A mixed analysis of variance with time (within: first half of the study vs. second half) and condition (between: *Matched* vs. *Unmatched*) revealed a trend toward an interaction of time with condition, *F*_(1, 12)_ = 4.29, *p* = 0.061. As shown in Figure 6, LSM scores increased slightly for children in the *Matched* condition (first: *M* = 0.66, *SD* = 0.25; second: *M* = 0.71, *SD* = 0.23; *d* = 0.21); the scores decreased slightly for children in the *Unmatched* condition (first: *M* = 0.74, *SD* = 0.23; second: *M* = 0.71, *SD* = 0.19; *d* = 0.14).

### 3.6. Language Emulation

As described earlier, phrase matching scores were computed against all previously heard stories, only stories from the same story scene, and only the story heard just prior to the child's. We used children's phrase matching scores as a measure of language emulation. We performed mixed analysis of variance with condition (between: *Matched* vs. *Unmatched*) and mean of sessions (within: sessions 1–4 vs. sessions 5–8) for the mean of children's exact and similar phrase matching scores per story and for the sum of children's exact and similar phrase matching scores across all stories.

#### 3.6.1. Compared to All Previously Heard Stories

**Figure 7:**
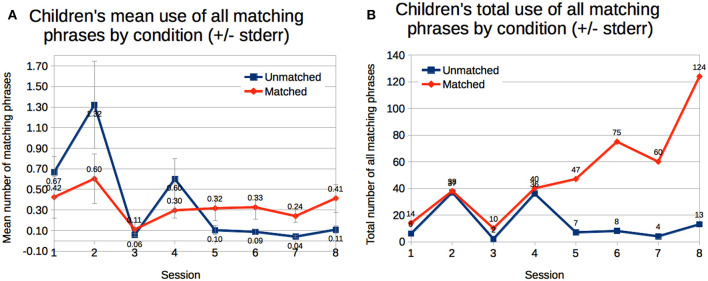
Children emulation the robot's phrases during their storytelling. Their emulation increased during the second half of the study in the *Matched* condition. **(A)** Children's emulation decreased in the *Unmatched* condition in the second half of the study. **(B)** Children's emulation increased in the second half of the study in the *Matched* condition.

We observed a trend for main effect of time on the mean number of matching phrases used per story, *F*_(1, 12)_ = 5.65, *p* = 0.035, and a significant interaction of time with condition, *F*_(1, 12)_ = 10.0, *p* = 0.008. Children emulated more of the robot's phrases per story in the first half of the study, and children in the *Unmatched* condition decreased usage more (Figure 7A). We observed a significant interaction of time with condition when looking at the sum of matching phrases across stories, *F*_(1, 12)_ = 9.81, *p* = 0.009. Children in the *Matched* condition increased their usage of matching phrases, while children in the *Unmatched* condition decreased their usage (Figure 7B).

#### 3.6.2. Compared to Stories Heard From the Same Story Scene

We observed a significant interaction of time with condition for the mean number of matching phrases used per story, *F*_(1, 12)_ = 9.10, *p* = 0.011. Children in the *Unmatched* condition used fewer matching phrases on average in the second half of the study, while children in the *Matched* condition did not change significantly. There were no significant differences for the sum of matching phrases across stories.

#### 3.6.3. Compared to the Story Heard Just Prior

We observed a trend for an interaction of time with condition for the mean number of matching phrases used per story, *F*_(1, 12)_ = 4.82, *p* = 0.048. Again, children in the *Unmatched* condition used fewer matching phrases in the second half of the study. There were no significant differences for the sum of matching phrases across stories.

### 3.7. Correlations

**Figure 8:**
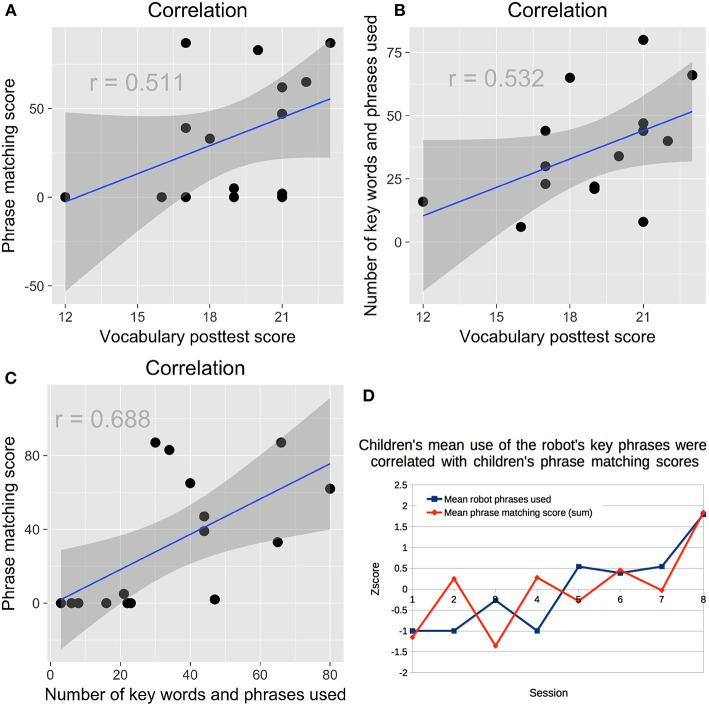
**(A)** Children who emulated more of the robot's phrases during their storytelling scored higher on the vocabulary posttest. **(B)** Children who used more of the robot's key words and phrases scored higher on the vocabulary posttest. **(C)** Children who emulated more of the robot's phrases were more likely to use the robot's key words and phrases. **(D)** Children's use of the robot's key words and phrases was correlated with their emulation of the robot's language over time.

Children who emulated more of the robot's phrases during their storytelling also scored higher on the vocabulary posttest, *r*_*s*15_ = 0.511, *p* = 0.052 (Figure 8A); as did children who used more of the robot's key words and phrases *r*_*s*15_ = 0.532, *p* = 0.041 (Figure 8B). Children who emulated the robot more during storytelling were also more likely to use more of the robot's key words and phrases, *r*_*s*15_ = 0.688, *p* = 0.003 (Figure 8C). This pattern was also apparent when looking at the mean of all children's scores for sessions 1–8 (Figure 8D).

**Figure 9:**
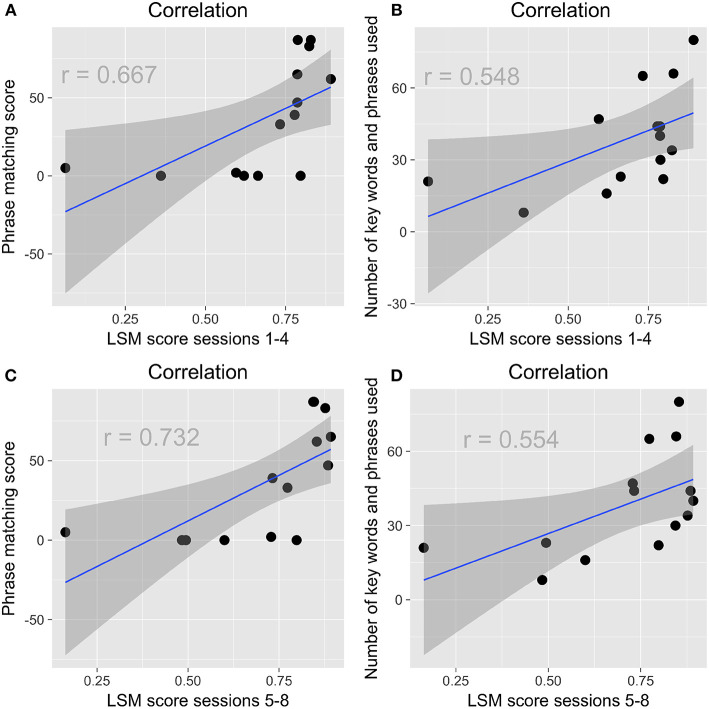
**(A)** In the first half of the study, children who had higher LSM scores were more likely to emulate the robot's phrases. **(B)** In the first half of the study, children who had higher LSM scores were more likely to use the robot's key words and phrases. **(C)** In the second half of the study, children who had higher LSM scores were more likely to emulate the robot's phrases. **(D)** In the second half of the study, children who had higher LSM scores were more likely to use the robot's key words and phrases.

Children who had higher LSM scores during sessions 1–4 were more likely to emulate the robot's phrases during storytelling, *r*_*s*15_ = 0.667, *p* = 0.007; they were also more likely to use the robot's key words and phrases, *r*_*s*15_ = 0.548, p = 0.034 (Figures 9A,B). The same pattern held for children's LSM scores in sessions 5–8 for phrase emulation, *r*_*s*14_ = 0.732, *p* = 0.003; and for key word and phrase use, *r*_*s*14_ = 0.554, *p* = 0.040 (Figures 9C,D). Children's LSM scores from sessions 1–4 were strongly correlated with their LSM scores from sessions 5–8, *r*_*s*14_ = 0.802, *p* < 0.001, suggesting little change in children's rapport and style matching over time.

**Figure 10:**
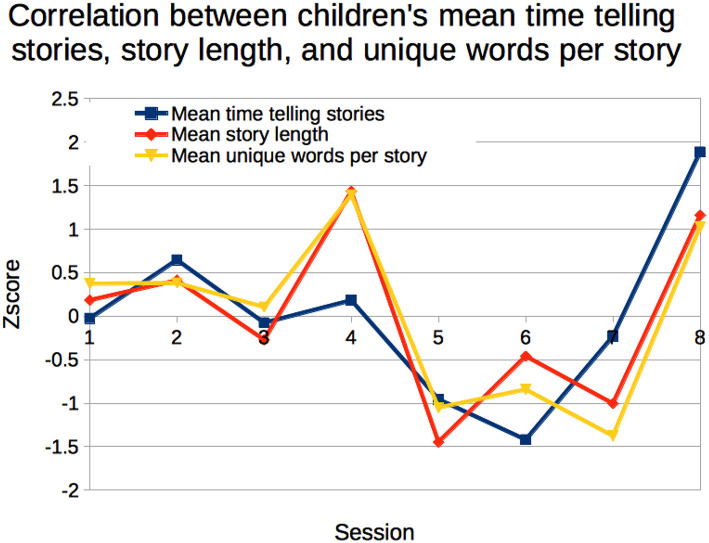
Children who told longer stories also used more unique words and spent more time telling their stories.

When looking at the mean of all children's scores for sessions 1–8, we observed that children who told longer stories also used more unique words (*r*_*s*8_ = 0.954, *p* < 0.001) and, as one might expect, spent more time telling their stories (*r*_*s*8_ = 0.715, *p* = 0.046; Figure 10).

## 4. Discussion

We asked whether children would show greater vocabulary learning and language emulation when they showed greater rapport with a social robot with whom they played a storytelling game over time. We found some evidence supporting our hypotheses.

First, we observed that most children liked the robot, and their LSM scores reflected that liking, being reasonably high overall. We observed that children learned new vocabulary words, as evidenced by higher vocabulary posttest scores and use of the target words in their stories. This result reflects prior work in which children have learned and mirrored new vocabulary words with social robots during storytelling activities (e.g., Kory Westlund et al., 2017b; Park et al., 2017a, 2019). However, because children were exposed to the target words during the pretest, it is possible that the pretest posed a first learning opportunity, and that they learned somewhat fewer words with the robot than the posttest indicates.

In partial support of H1, we observed that children's LSM scores were positively related to their use of the robot's key words and phrases. However, contrary to our expectations, LSM scores were not significantly related to children's vocabulary test scores.

This may be for several reasons. First, because the sessions with the robot were fairly short (10–15min) and because not all children told long stories, the amount of conversation between the robot and child was limited. As such, the amount of data used to compute the LSM scores was limited, and the LSM scores should be interpreted with a degree of caution. Second, children's LSM scores may not perfectly reflect rapport. Prior work linked higher LSM scores between two people to higher rapport and a deeper relationship (e.g., Pennebaker et al., 2003; Ireland et al., 2011; Babcock et al., 2014), but this work has primarily been done with adults, not children. Third, we do not know exactly how rapport affects learning, and thus, the causal connection between rapport and learning seen in earlier work in human-human peer tutoring (Sinha and Cassell, 2015a,b) may not appear with younger children in a language learning context. Rapport may not necessarily directly impact learning; it may be, for example, that rapport increases emulation of various behaviors, which in some contexts could increase learning, or that rapport facilitates being in a more positive state of mind, which perhaps leads to more engagement and learning. Furthermore, rapport may play a different role in peer learning with social robots than in other contexts with humans.

In our analyses here, we did observe that children's LSM scores correlated positively with their emulation of the robot during storytelling, as expected (H2). This suggests that rapport is linked to emulation, which is in line with prior work showing that people will mirror a variety of different behaviors in others with whom they have high rapport (e.g., Tickle-Degnen and Rosenthal, 1990; Chisholm and Strayer, 1995; Dijksterhuis and Bargh, 2001; Rotenberg et al., 2003; Dijksterhuis, 2005; Chartrand and van Baaren, 2009; Wiltermuth and Heath, 2009; Lubold, 2017).

In addition, we saw that children's emulation of the robot's language was positively correlated with their vocabulary scores, supporting H3. Children who correctly identified more of the target words on the receptive vocabulary test were also more likely to expressively use the words in their stories. These results suggest that children's emulation was related to their learning—perhaps their rapport with the robot led to greater emulation, and greater emulation was indicative of greater word learning. This would be worth investigating in a systematic way in follow-up work.

We find partial support for H4: When examining children's behavior over time, we saw that children slightly increased their use of the robot's keywords and phrases from the first half of the study to the second half. However, children's overall emulation decreased over time, while their use of unique words increased. It may be that children were more creative over time when telling stories, making up their own that drew less on the robot's stories for inspiration. The storytelling activity was designed to facilitate language development, so both creatively using language as well as imitating the robot's language were beneficial outcomes. Story re-telling (i.e., intentionally imitating another's storytelling) has often been used as an educational activity for helping children learn stories and vocabulary (e.g., Isbell, 2002; Dunst et al., 2012; Kory Westlund et al., 2017b; Otwinowska et al., 2018; Kory-Westlund and Breazeal, 2019b).

Children's LSM scores, on average, did not show a strong increase over time (there were differences by condition, as discussed further below). This could indicate little increase in rapport, or could mean that LSM is not sufficiently sensitive to capture children's changes in rapport over the study.

Children's LSM scores and phrase emulation during storytelling increased over time for children in the *Matched* condition, but decreased slightly for children in the *Unmatched* condition. Children in the *Matched* scoring also had higher scores on the vocabulary posttest. These results provide some support for H5; however, given the small sample size, these results should be interpreted with caution. The robot's story level personalization appeared to positively impact children's emulation of the robot's language, their rapport as indexed by LSM, and their vocabulary learning. This is in line with prior work showing links between a robot's personalization and children's engagement and learning (e.g., Leite et al., 2012; Gordon et al., 2016; Palestra et al., 2016; Scassellati et al., 2018; Park et al., 2019)

However, in addition to the small sample size, the two conditions were not fully balanced. There were more children in the *Matched* condition and there was only one boy in the *Unmatched* condition. In addition, although children were assigned to conditions prior to the start of the robot interaction using their initial language assessment scores to attempt to balance language ability across conditions, we did observe somewhat higher scores for children in the *Unmatched* condition across various metrics during the first half of the study (prior to the robot's personalization/matching, which only occurred in the second half of the study). We expect that were the groups more balanced, these initial differences may be smaller or might even disappear, while differences between conditions as a result of the personalization would be larger.

Taken together, our results suggest that first, interacting with a more advanced peer-like social robot can be beneficial for children's language learning. This is in line with work examining children's language learning with human peers (Fuchs et al., 1997; Mathes et al., 1998; Topping, 2005; Schechter and Bye, 2007; Whitebread et al., 2007; Mashburn et al., 2009; Justice et al., 2011; DeLay et al., 2016; Lin et al., 2016). Second, children's emulation of the robot's language may be related to their rapport and to their learning. Earlier work has shown that children will emulate the behavior of social robots—including mirroring expressiveness (Spaulding et al., 2016), curiosity (Gordon et al., 2015), and language (Kory Westlund et al., 2017b)—but had not yet explored mechanisms that might affect children's emulation and peer learning. Our results suggest that rapport may be one such mechanism. This is the first study we know of to empirically support that rapport may indeed be a modulating factor in children's peer learning.

Finally, this study highlights new opportunities we have for using social robots as interventions for early language development, specifically by leveraging this connection between rapport and learning.

### 4.1. Limitations

This study had several limitations. First, as mentioned earlier, the sample size was fairly small and conditions were unbalanced in number. As such, the statistical power of our analyses are underpowered. In addition, children's individual differences were not controlled for, such as learning ability or socio-economic status. These factors may all influence children's learning and social interactions with the robot. Future work should attempt to recruit a more balanced, homogeneous sample and explore the stability of the results across individual differences.

The target vocabulary words presented in the robot's stories included some words that were known by numerous children at the start of the study (as reported above, children identified a mean of 13.4 of 24 words correctly at the pretest, *SD* = 3.62). The difference between children's vocabulary scores on the pretest vs. the posttest did show that children knew more of the words at the end of the study, but because a set of common words and not nonce words were used, we cannot know for sure that children learned these words as a result of the robot interaction or because of other events that occurred during the two months during which the study took place.

Another limitation of the dataset was the lack of additional assessments of relationship and rapport. We used children's LSM scores as a measure of rapport, since numerous prior studies have linked higher LSM scores between two people to higher rapport and a deeper relationship (e.g., Pennebaker et al., 2003; Ireland et al., 2011; Babcock et al., 2014). However, future work should endeavor to measure children's rapport and relationship with the robot in additional ways, e.g., using measures presented in Kory-Westlund et al. (2018) and Kory-Westlund and Breazeal (2019a).

Finally, this study explored a one-on-one interaction with the robot. However, children often learn with others—friends, siblings, parents, and teachers. Future work should explore group interactions that include multiple children or children with parents, caregivers, and teachers. This could give us insight into how to integrate robots into real-world educational contexts, such as schools and homes.

Despite these limitations, we did see numerous correlations and differences that are suggestive of links between children's learning, rapport, and language emulation. While these results are exploratory and not definitive, they do provide evidence that this in an area that warrants further study.

## Data Availability

The datasets generated for this study are available on request to the corresponding author.

## Author Contributions

JK-W and CB: the study was conceived, designed, the paper was drafted, written, revised, and approved. JK-W: data analysis was performed.

### Conflict of Interest Statement

The authors declare that the research was conducted in the absence of any commercial or financial relationships that could be construed as a potential conflict of interest.
